# Incidental sternal foramen discovered during the evaluation of a traumatic fracture: a case report

**DOI:** 10.1093/jscr/rjaf299

**Published:** 2025-05-12

**Authors:** César L Baute, Daniela Saenz

**Affiliations:** Department of Medical Research, Universidad Francisco Marroquín, 6a. Calle Final, Calle Manuel F. Ayau, Zona 10, Guatemala City 01010, Guatemala; Department of Medical Research, Universidad Francisco Marroquín, 6a. Calle Final, Calle Manuel F. Ayau, Zona 10, Guatemala City 01010, Guatemala

**Keywords:** sternal fracture, sternal foramen, open reduction, thoracic trauma

## Abstract

Traumatic sternal fractures are rare injuries, typically caused by high-energy blunt chest trauma. While most cases are managed conservatively, the presence of congenital anomalies, such as a sternal foramen, can complicate diagnosis and treatment. This case report presents a 39-year-old male with severe retrosternal pain after a motorcycle collision. Imaging revealed a non-displaced oblique sternal fracture adjacent to a sternal foramen. Due to pain severity and fracture location, open reduction and internal fixation (ORIF) was performed with precautions to avoid mediastinal injury. This case highlights the importance of detailed imaging to identify anatomical variations that may influence management. ORIF provided effective stabilization, pain relief, and minimized the risk of complications in the presence of a sternal foramen.

## Introduction

Traumatic sternal fractures are relatively uncommon in blunt thoracic trauma, accounting for a small proportion of cases, with the majority occurring because of high-energy impacts [1]. The mid-body of the sternum is the most frequently affected region due to its anatomical prominence [2]. While most cases are managed conservatively, certain anatomical variations, such as congenital sternal anomalies, may necessitate surgical intervention.

A sternal foramen, a congenital defect caused by incomplete ossification, occurs in approximately 4.3% to 13.8% of individuals [3]. Though generally asymptomatic, this condition can increase the risk of mediastinal injury during trauma or invasive procedures, exposing critical structures like the pericardium and major vessels [4]. Advanced imaging, particularly 3D computed tomography (CT), is essential for identifying both fractures and congenital anatomical anomalies.

This report describes a 39-year-old male with a traumatic sternal fracture adjacent to a congenital sternal foramen, emphasizing the importance of detailed imaging and careful surgical planning.

## Case report

A 39-year-old male was admitted to the emergency department following a motorcycle accident. He presented with a one-day history of severe, non-radiating, stabbing retrosternal pain, which was unrelieved by analgesics. The pain worsened with deep inspiration and progressively intensified over the next 24 hours. On examination, vital signs were stable. Inspection revealed mild erythema in the sternal region with localized tenderness on palpation, but no crepitus or signs of thoracic instability. No respiratory distress or auscultatory abnormalities were noted, and the remainder of the physical examination was unremarkable.

**Figure 1:**
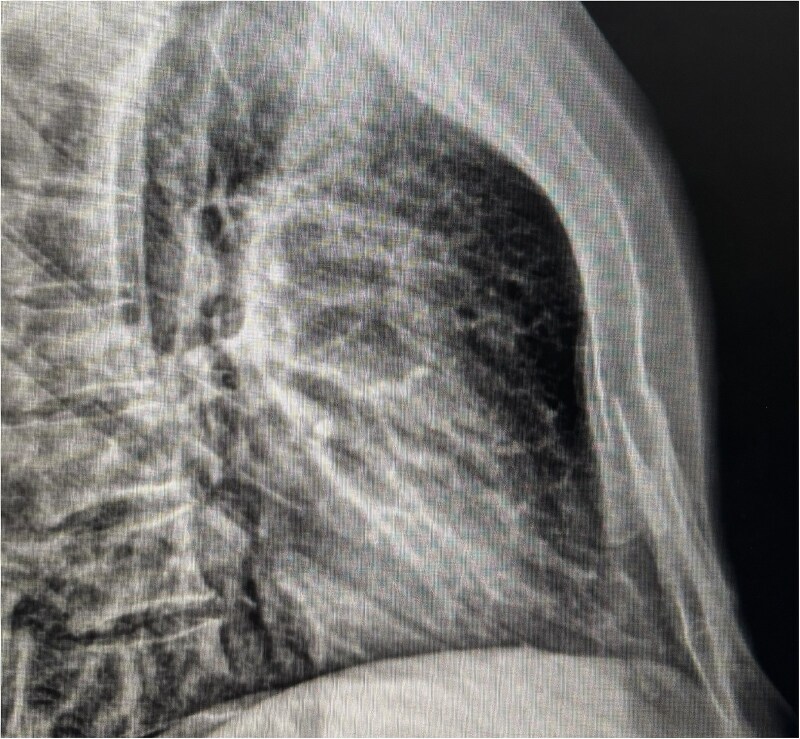
Lateral chest X-ray showing cortical discontinuity in the midportion of the sternal body (red arrow), indicative of an oblique fracture.

**Figure 2:**
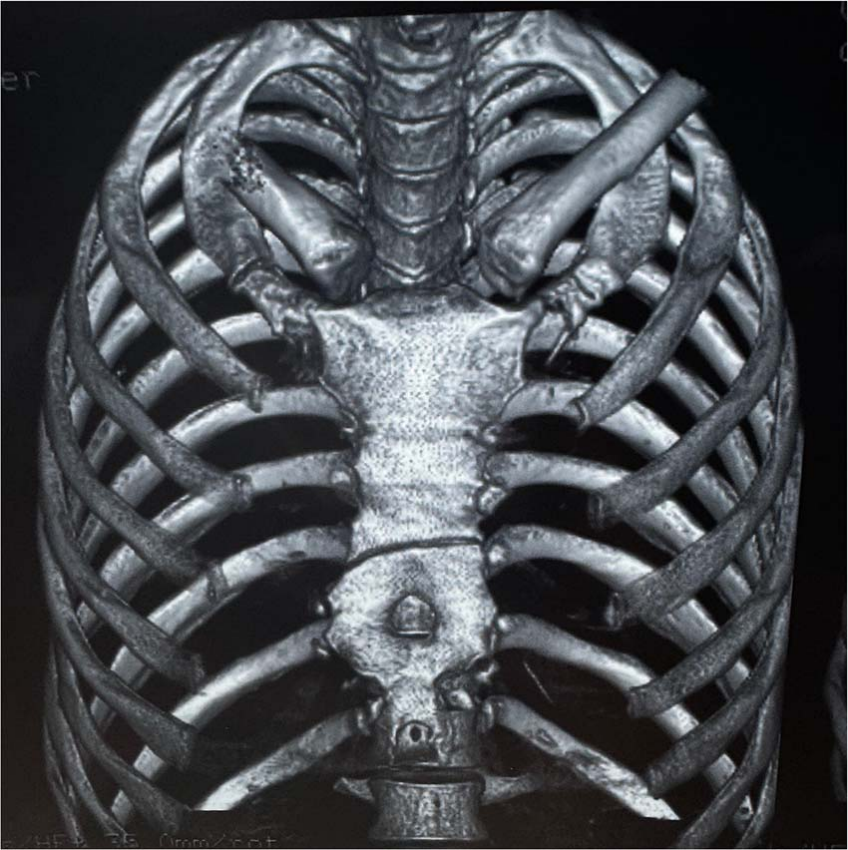
CT (3D reconstruction) revealing a non-displaced sternal fracture and the sternal foramen adjacent to the fracture site.

**Figure 3:**
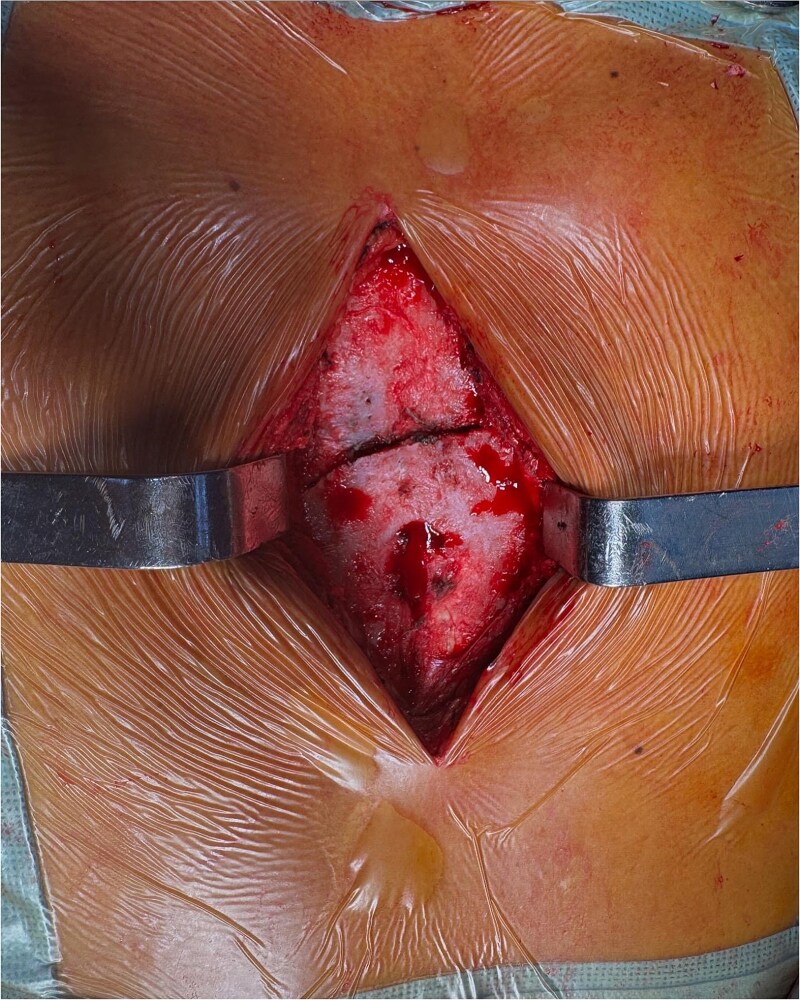
Intraoperative image showing exposure of the sternal fracture before fixation, with the sternal foramen identified in the lower region.

Chest radiographs demonstrated an oblique fracture of the mid-sternum, indicated by a discontinuity in the anterior and posterior cortical surfaces (Fig. 1). Additionally, a 3D CT scan confirmed the presence of a non-displaced oblique fracture (Fig. 2) and revealed a sternal foramen adjacent to the fracture site (Fig. 3). This anatomical variation raised concerns of a potential mediastinal injury in the event of fracture displacement or during surgical intervention.

**Figure 4:**
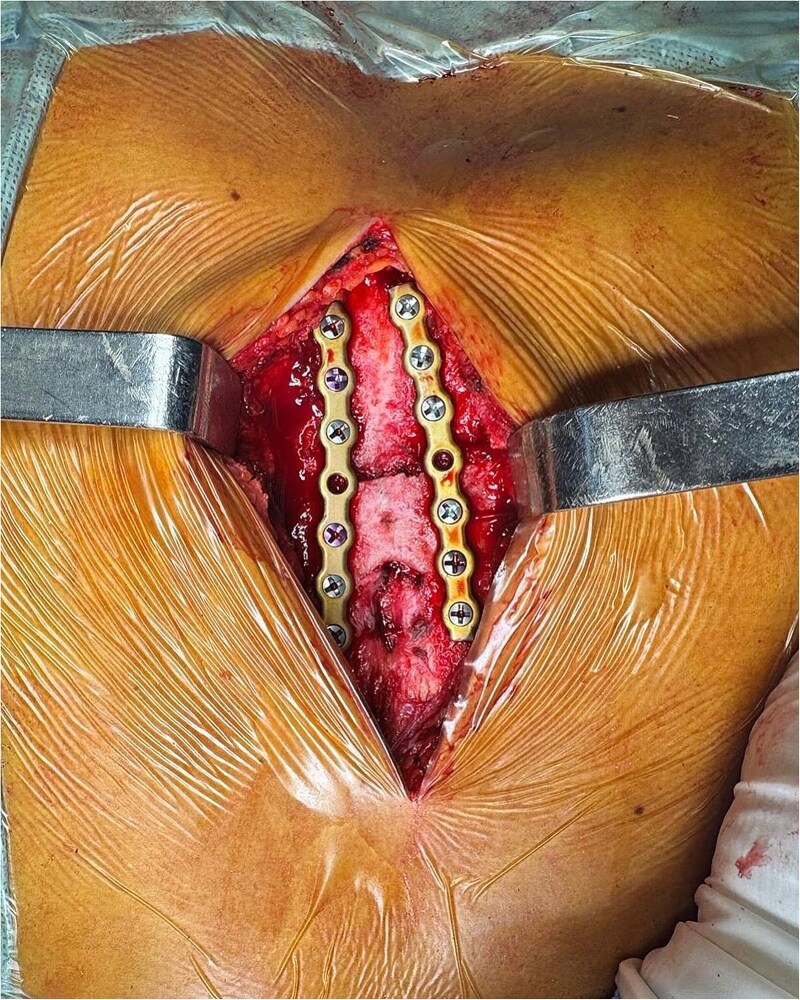
Final fixation after placement of two parallel 7-hole plates on the sternal body.

Due to the severity of pain and functional limitations, as well as the proximity of the fracture to the sternal foramen, open reduction and internal fixation (ORIF) was performed. During the procedure, the sternal fracture was exposed, and the sternal foramen was identified near the fracture site (Fig. 4). Two parallel 7-hole plates were placed on the anterior surface of the sternum to stabilize the fracture. Intraoperative precautions, including real-time imaging and meticulous dissection, were employed to avoid injury to underlying mediastinal structures.

Postoperatively, the patient experienced significant pain relief and regained thoracic mobility. Chest radiographs confirmed proper placement of the plates, with no signs of displacement or complications. The patient was discharged after 48 hours with instructions for outpatient follow-up and temporary restriction of intense physical activity. At two weeks, the patient reported minimal pain and showed adequate bone consolidation on imaging.

## Discussion

Sternal fractures represent ~3% to 8% of closed thoracic trauma cases and, while rare, can lead to serious complications if not properly diagnosed and treated [1]. High-energy trauma, including motor vehicle accidents and falls from heights, is the most common cause of these injuries. The clinical diagnosis of sternal fractures can be challenging, as symptoms may overlap with other thoracic pathologies. The hallmark of sternal fractures is retrosternal pain, which is typically aggravated by deep inspiration [1]. While lateral chest radiographs may suggest the presence of a sternal fracture, CT remains the gold standard for confirming the diagnosis, assessing the extent of the fracture, and detecting associated injuries [2].

In this case, the CT scan not only confirmed the presence of a non-displaced oblique fracture but also revealed the presence of a sternal foramen adjacent to the fracture. A sternal foramen is a congenital defect arising from incomplete ossification during embryonic development. Its prevalence ranges from 4.3% to 13.8% [3]. Though typically asymptomatic, a sternal foramen can increase the risk of complications during invasive thoracic procedures or direct trauma to the chest [4]. When present in conjunction with a traumatic sternal fracture, the proximity of the foramen to the fracture line poses a risk to the underlying mediastinal structures, which may be unprotected around the bone defect [3, 4].

A sternal foramen results from incomplete fusion of the sternal ossification centers during embryogenesis. While most cases remain clinically silent, this anatomical defect can have significant implications in trauma and surgery. The presence of a sternal foramen may lead to increased vulnerability of mediastinal structures, such as the pericardium, great vessels, and thymus, especially in cases of penetrating injuries or fractures that extend through the defect [5, 6]. This increases the risk of life-threatening complications, particularly if the foramen is large or positioned near critical structures. Additionally, unrecognized sternal foramina have been associated with inadvertent cardiac punctures during procedures such as sternal bone marrow aspiration, acupuncture, and median sternotomy [6].

The management of sternal fractures complicated by a sternal foramen requires careful surgical planning. Typically, non-displaced fractures without respiratory compromise are managed conservatively with pain control and rest [7, 8]. However, certain anatomical and clinical factors, such as the proximity of a sternal foramen to the fracture site, may warrant surgical intervention. Studies have demonstrated that sternal fixation provides significant benefits over conservative treatment, including improved pain control, reduced risk of deformities, and better long-term quality of life outcomes [9]. In patients with anatomical risk factors, surgical fixation helps prevent complications such as fracture displacement or mediastinal injuries.

Intraoperative considerations are crucial when dealing with a sternal foramen during surgery. Given the potential absence of a bony barrier protecting mediastinal structures, surgeons must exercise extreme caution when performing sternal fixation or reconstructive procedures [10]. The use of preoperative imaging, including 3D CT reconstruction, is essential to accurately map the location of the foramen and plan surgical approaches accordingly. When necessary, intraoperative fluoroscopy or ultrasonography can provide additional guidance to minimize the risk of inadvertent injury [11]. In cases where a large foramen compromises structural integrity, bone grafting or mesh reinforcement may be considered to provide additional support and reduce the risk of long-term complications such as chronic pain or instability [5].

This case highlights the value of a multidisciplinary approach to managing complex sternal fractures. Recognizing congenital anatomical variations is vital in the management of thoracic trauma and surgical interventions. While often asymptomatic, the presence of this defect can significantly alter clinical decision-making in cases of trauma, fractures, and planned surgical procedures. A thorough preoperative evaluation, careful surgical technique, and multidisciplinary collaboration remain key factors in ensuring optimal patient outcomes.

## Conclusion

This case underscores the complexities involved in diagnosing and managing traumatic sternal fractures, particularly when complicated by anatomical variations such as a sternal foramen. While sternal fractures are rare, they can lead to significant complications if not properly identified and treated. The presence of a sternal foramen in the context of a traumatic fracture necessitates careful surgical planning to avoid damage to underlying mediastinal structures. ORIF proved to be an effective treatment option, ensuring pain relief, stable fracture fixation, and minimizing the risk of long-term complications. A multidisciplinary approach, involving thoracic surgeons, radiologists, and anesthesiologists, was crucial to achieving a successful outcome and highlights the importance of comprehensive preoperative assessment and collaborative care in managing these challenging injuries.
